# Detrimental Impact of Microbiota-Accessible Carbohydrate-Deprived Diet on Gut and Immune Homeostasis: An Overview

**DOI:** 10.3389/fimmu.2017.00548

**Published:** 2017-05-12

**Authors:** Claire Immediato Daïen, Gabriela Veronica Pinget, Jian Kai Tan, Laurence Macia

**Affiliations:** ^1^Nutritional Immunometabolism Node Laboratory, Charles Perkins Centre, The University of Sydney, Sydney, NSW, Australia; ^2^School of Medical Sciences, The University of Sydney, Sydney, NSW, Australia; ^3^Institut de génétique moléculaire de Montpellier, UMR5535, Montpellier University, Montpellier, France; ^4^Infection and Immunity Program, Department of Biochemistry and Molecular Biology, Monash Biomedicine Discovery Institute, Monash University, Clayton, VIC, Australia

**Keywords:** dietary fiber, microbiota-accessible carbohydrates, immunity, gut homeostasis, gut barrier, gut microbiota, epithelium

## Abstract

Dietary fibers are non-digestible polysaccharides functionally known as microbiota-accessible carbohydrates (MACs), present in inadequate amounts in the Western diet. MACs are a main source of energy for gut bacteria so the abundance and variety of MACs can modulate gut microbial composition and function. This, in turn, impacts host immunity and health. In preclinical studies, MAC-deprived diet and disruption of gut homeostasis aggravate the development of inflammatory diseases, such as allergies, infections, and autoimmune diseases. The present review provides a synopsis on the impact of a low-MAC diet on gut homeostasis or, more specifically, on gut microbiota, gut epithelium, and immune cells.

## Introduction

In recent decades, the prevalence of non-communicable diseases, such as allergies, autoimmune, and inflammatory diseases has increased drastically in Western lifestyle countries. For instance, the incidence of asthma in Swedish schoolchildren doubled between 1979 and 1991 (1), the incidence of multiple sclerosis in Germany (Lower Saxony) doubled from 1969 to 1986 (2), and the incidence of Crohn’s disease in northern Europe more than tripled from the 1950s to the 1990s (3).

Although largely unexplained, these increases are likely to have a strong environmental component (4, 5). Dramatic lifestyle changes followed the industrial revolution, among which a nutritional transition from a traditional diet to the Western diet (6). This diet consists of heavily processed foods, rich in fat, sugar, protein, and a variety of additives, while remaining low in micronutrients and dietary fiber (7). While most nutrients are absorbed in the duodenum during the digestion process, dietary fiber remains intact until it reaches the colon, which is inhabited by trillions of bacteria known as the gut microbiota (8, 9). Dietary fibers are complex carbohydrates of plant origin, broken down by specialized enzymes produced by gut bacteria but indigestible by the host (10). They have recently been redefined as microbiota-accessible carbohydrates (MACs) and represent the major energy source for colonic bacteria (11). The recommended daily intake of dietary fiber is at least 30 g, although, on average, those on the Western diet only consume 15 g (7). In a cohort of 219,123 men and 168,999 women, Park et al. studied the association between intake of dietary fiber, ranging from 11 to 29 g/day, and death from cardiovascular, infectious, and respiratory diseases, with a 9-year follow-up (12). This study shows that dietary fiber intake was significantly associated with a 22% decrease in mortality rate in both genders (multivariate relative risk comparing the highest with the lowest quintile). Areas described as “food deserts,” defined by the absence of healthy food availability in a one mile radius, are characterized by low-MAC consumption and an increased incidence of asthma in children (13).

People in traditional societies, where fiber intake can reach 50–120 g/day, are associated with a much more diverse gut microbiota when compared with people in Western countries (14–16). A diverse microbiome is associated with “good health,” while low diversity and dysbiosis have been correlated to diseases highly prevalent in Western society, such as obesity, type 2 diabetes, inflammatory bowel diseases, rheumatoid arthritis, and asthma (17–20).

Short-chain fatty acids (SCFAs), namely, acetate, butyrate, and propionate, are released by gut bacteria during fermentation of dietary fibers. These can bind to the G protein-coupled receptors such as GPR41, GPR43, and GPR109a that are expressed by both immune and non-immune cells (4, 9, 21, 22). They are also known to inhibit histone deacetylases (HDACs) activity in immune cells, promoting anti-inflammatory cells (5, 23). The immune effects of SCFAs are further detailed in Section “Impact of Low Dietary MACs on Immune Cells and Disease Development.” SCFAs play a key role in health and disease and are found at lower concentrations in populations consuming a Western diet when compared to those consuming a traditional diet (14). Therefore, low bacterial diversity, as well as low SCFAs, could partially explain the rise of non-communicable diseases in Western countries (24, 25).

**Figure 1:**
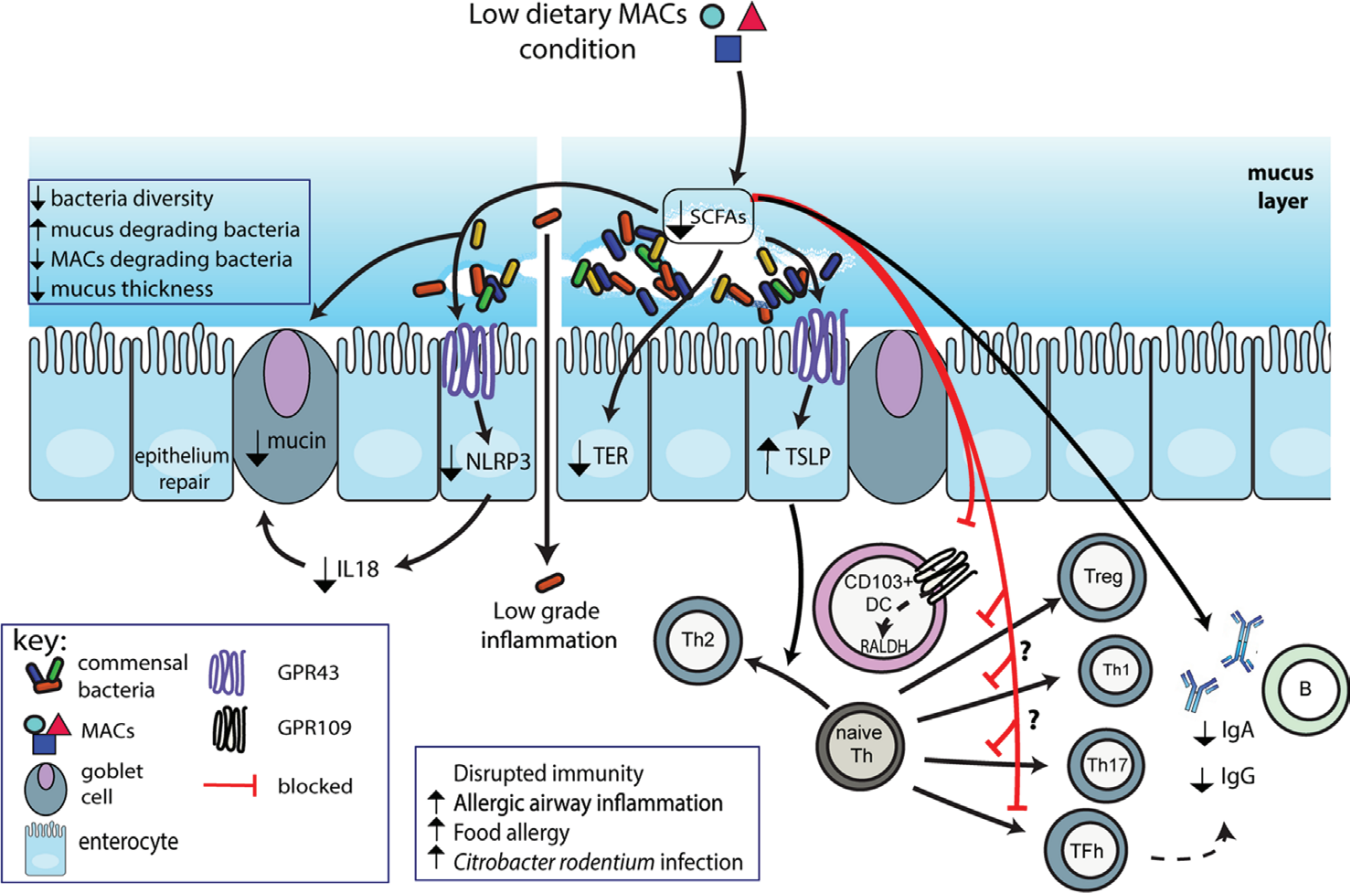
**Schematic representation of impact of low dietary microbiota-accessible carbohydrates (MACs) on microbiota and gut homeostasis**. Consumption of low dietary MACs leads to decreased gut bacterial diversity with outgrowth of mucus-degrading bacteria and decreased levels of MAC-degrading bacteria. The mucus layer will thus become thinner exacerbated by reduced production of Muc2 due to reduced short-chain fatty acids (SCFAs) production. This decrease in production of SCFAs also impairs GPR43 activation on epithelial cells leading to increased production of proTh2 cytokine TSLP and decreased activation of NLRP3, and thus, decreased production of epithelial healing cytokine IL-18. Low SCFAs also impair epithelial barrier function as shown by decreased transepithelial resistance (TER), leading to increased bacterial product translocation into the lamina propria, triggering inflammatory reactions. Finally, due to their key role in immune function, low SCFAs impair regulatory T cell (Treg), Th1 cell, and Th17 cell, while enhancing T helper 2 (Th2) development. Decreased direct activation of B-cells and indirect activation through decreased T follicular helper cells impair IgA and IgG production.

Microbiota-accessible carbohydrates are the main source of carbon for colonic bacteria and favor an increase in beneficial bacteria. The composition and function of the gut microbiota is thus highly dependent on the availability of MACs. While the beneficial impact of supplementation with dietary MACs on gut microbiota, host metabolism, and immunity has been reported before, the present review will focus on the deleterious impact of low-MAC consumption on gut homeostasis, immunity, and disease development as illustrated in Figure 1.

## Impact of Low MACs on GUT Microbiota

Diet composition, particularly the availability of MACs, has a major impact on gut microbiota.

### Low Dietary MACs Promote Mucus-Degrading Bacteria

Depending on their enzymatic machinery, gut bacteria can either extract carbon from dietary MACs or endogenous mucus as source of energy (26). The mucus layer is produced by specialized epithelial cells, the goblet cells and is characterized by a glycoprotein-rich layer that overlies the gut epithelium. This layer represents the first line of defense against commensal and pathogenic bacteria (27). Two subsets of gut bacteria can degrade the mucus, either bacteria extracting their energy exclusively from the mucus: the mucin-degrading specialists (i.e., *Akkermansia muciniphila*) or from both the mucus and dietary MACs: the mucin-degrading generalists (i.e., *Bacteroides caccae*) (28). Under low dietary MAC conditions, mucin-degrading generalists shift from utilization of dietary polysaccharides to mucus glycan (28). It was recently shown that MAC deficiency leads to a rapid increase in abundance of mucin-degrading bacteria (both mucin-degrading specialists and generalists) and a decrease in MAC-degrading species (29). Transcriptomic analysis revealed that MAC availability in the diet could modulate the expression of glycoside hydrolases and polysaccharide lyases, enzymes specialized in complex polysaccharide digestion (26). Under low dietary MAC conditions, these mucus-degrading enzymes are upregulated and *vice versa*.

Some bacteria have also evolved ways to extract and release polysaccharides from their own cell wall through the activation of outer surface glycoside hydrolases. These membrane polysaccharides can then become available for surrounding bacteria, in a mechanism called cross-feeding (30). *Bacteroides ovatus*, an important MAC-degrading bacterium, is a crucial source of nutrients for other bacteria, *via* cross-feeding (30). Hence, dietary MAC content will not only impact mucus or MAC-dependent bacteria but also the growth of such cross-fed bacteria.

The microbial changes observed in studies using MAC-restricted diets and described in this review (4, 5, 22, 26, 29, 31, 32) are summarized in Table 1. The most consistent changes observed in a low-MAC diet compared to a high-MAC diet, are an increase in Proteobacteria and a decrease in Bacteroidetes, as well as a decline in bacterial diversity.

**Table 1:** **Microbiota changes in mice fed on low dietary microbiota-accessible carbohydrates (MACs)**.

Studies	Mice studied	Method used for gut analysis	Low-MAC diet	Comparator diet	Changes observed in low-MAC diet group	
					Decrease	Increase
Desai et al. (29)	Germ-free Swiss Webster mice colonized by synthetic human microbiota	Illumina sequencing of 16S rRNA genes (V4 region)	0% fiber. Starch and maldodextrin replaced with glucose (Harlan TD.140343)	High MACs: 4.2% crude fiber (14.6% ND fiber; 5.3% AD fiber) (LabDiet 5010)	Fiber-degrading species: *Bacteroides ovatus*; *Eubacterium rectale*	Mucin specialists (*Akkermansia muciniphila*; *Barnesiella intestinihomnis*) and mucin generalists (*Bacteroides thetaiotaomicron*; *Bacteroides caccae*)
Sonnenburg et al. (26)	Germ-free Swiss Webster mice colonized by human microbiota	Illumina sequencing of 16S rRNA genes (V4 region)	Carbohydrates from sucrose (31%), corn starch (31%), and cellulose (5%) (Harlan TD.86489)	High-MAC (LabDiet 5010)	Diversity (Shannon index), Bacteroidales sp.	Clostridiales
Macia et al. (4)	C57BL/6 mice	Roche 454 sequencing of 16s rRNA gene (V2–V3 region)	Modification of AIN-93G. Fiber, starch, and dextrinized starch replaced by dextrose (SF09-028) devoid of fiber or starch	High MACs: modification of AIN-93G enriched in guar gum (20%) and cellulose (20%) (SF11-029)	Prevotellaceae family	Bacteroidaceae; *Oscillibacter* sp.
Thorburn et al. (5)	C57Bl6 and BALB/c mice	Roche/454 FLX sequencing of 16S rRNA genes (V1–V3 region)	Crude fiber 0%; AD fiber 0% (SF09-028)	High MACs Gel crisp starch. Crude fiber 3.2%; AD Fiber 4.2% (SF11-025)	Diversity (Shannon index), observed species and equability (chao1); Bacteroidetes, Bacterioidaceae, and Bacteroidales	Proteobacteria phylum, especially: *Pandoraea*, Burkholderiaceae
Tan et al. (22)	C57BL/6 mice	Illumina sequencing of 16S rRNA genes (V3–V4 region)	Modification of AIN-93G devoid of fiber or starch (SF09-028)	High MACs (SF11-029)	Proteobacteria, Deltaproteobacteria, and Desulfovibrionales	Firmicutes bacilli, Lactobacillales
Kim et al. (31)	C57BL/6 mice	qPCR analysis of 16S rRNA gene sequences	0% fiber	High MACs: 15% of pectin and inulin (1:1)	Bacteroidetes phylum	Proteobacteria phylum
Trompette et al. (32)	C57BL/6 female mice	Roche/454 FLX sequencing of 16S rRNA genes (V1–V3 region)	<0.3% fiber (Provimi Kliba diet 2122)	Normal chow 4% fiber (Provimi Kliba diet 3202)	Diversity (Shannon index) and richness (operational taxonomic units)	Proteobacteria phylum

*AD, acid detergent; ND, neutral detergent*.

### Low Dietary MACs Lead to Decreased Bacterial Diversity with Irreversible Loss of Bacterial Strains

A recent breakthrough has been made in the impact of low dietary MACs on gut microbiota (26). Mice with a humanized microbiota, fed on a low-MAC diet for 7 weeks, had significantly decreased bacterial diversity, which was never fully restored despite switching their diet to a high-MAC diet for the following 15 weeks. This phenomenon is described as “scars” of the microbiota, characterized by the disappearance of specific operational taxonomic units, while other bacteria are more resilient and able grow back to their initial levels. Over three generations on low-MAC feeding, the gut microbiota was stably “dysbiotic,” with a decline in taxa, mostly of Bacteroidales, and a marked loss of the glycoside hydrolase repertoire, the enzymes necessary to process MACs. By the fourth generation on low-MAC feeding, a high-MAC diet was fully inefficient at restoring bacterial diversity, which could only be overcome by the administration of high-MAC microbiota in addition to the high-MAC diet. The Western diet is not only deprived of MACs but also has macronutrient imbalance and micronutrient deprivation, which suggests that in humans, stable dysbiosis over three to four generations could be the best-case scenario, although quicker irreversible dysbiosis may be closer to reality. While this study did investigate the impact of such a diet on disease development, we also demonstrate that reconstitution of germ-free mice with microbiota from mice fed on an MAC-deprived diet was associated with more severe colitis and food allergy (4, 22). This suggests that dysbiotic microbiota due to low MACs can exacerbate non-communicable disease development.

## Impact of Low Dietary MACs on GUT Epithelium

The gut epithelium is a dynamic barrier ensuring the physical separation between the host and the gut microbiota while also enabling their communication (33). Epithelial integrity is a pillar of both gut and overall health. Indeed, increased gut permeability, linked to Western diet consumption and dysbiosis, facilitates translocation of bacterial products in the host (8). This might be a trigger for allergic, autoimmune, or endocrine diseases (25).

### Low Dietary MACs Contribute to Decreased Epithelial Integrity

Bacterially derived SCFAs ensure epithelial integrity by increasing transepithelial resistance (34), as well as promoting mucus secretion (35). SCFAs increase prostaglandin secretion by subepithelial myofibroblasts, which, in turn, promote epithelial mucin expression, a key component of the mucus layer (36). Butyrate, but neither acetate nor propionate, upregulated colonic mucin expression *in vitro*, by inhibiting HDAC activity (37). SCFAs have also been recently found to be critical for epithelium repair, through the direct activation of NLRP3 inflammasome in gut epithelial cells, leading to release of IL-18 (4). NLRP3 inflammasome activation is protective in a murine model of colitis induced by dextran sodium sulfate (DSS) (38). Mice fed on an MAC-deprived diet had significantly less IL-18, and worse DSS-induced colitis (4, 39). Inflammasome activation is a two-step process involving both a priming and a trigger phase (39). While these phases are well characterized in macrophages, they remain elusive in epithelial cells. It was recently shown that fecal extracts, containing various microbial associated molecular patterns (MAMPs) could prime the NLRP3 inflammasome, and that acetate could trigger this pathway by inducing cell hyperpolarization secondary to increased intracellular Ca^2+^. Interestingly, fecal supernatants from low MAC-fed mice were less potent in priming NLRP3 inflammasome compare to those from high MACs probably due to the different MAMPs present (4). While most studies focus on SCFAs as critical in promoting beneficial effects of MACs in diseases, it is important to note that beneficial changes in the microbiota by itself is also critical, as treatment with SCFAs in drinking water only partially recapitulates the effects of MACs (4, 22).

Microbiota-accessible carbohydrate-induced microbiota changes can also directly impact epithelial integrity. As described above, under low-MAC conditions, some bacteria use the mucus layer as a source of energy. This phenomenon thins the mucus layer and impairs the epithelial barrier. This low MAC-induced gut permeability was associated with increased susceptibility to the epithelium-targeting pathogen *Citrobacter rodentium* suggesting that dietary MACs are key in fighting gastrointestinal infections (29).

### Low Dietary MACs Modify Epithelium Cytokine Expression

While MACs have the ability to affect the bacterial–epithelium interaction, they can also affect the epithelial–immune interaction (22, 40). Mice fed on a low-MAC diet had increased expression of epithelial *tslp*, a cytokine known to direct the immune response toward T helper 2 (Th2), involved in allergy development. On the other hand, high MACs, through release of SCFAs and subsequent activation of GPR43 in the epithelium, had the opposite effect (22). Moreover, low IL-18 mediated by low-MACs, might affect immune cells such as natural killer cells, which remain unexplored.

Altogether, these results illustrate the detrimental impact of a low-MAC diet on the gut microbiota–epithelium–immune axis.

## Impact of Low Dietary MACs on Immune Cells and Disease Development

This section highlights several key studies examining the opposing effects of MAC deprivation vs. enrichment on the immune system. Diet composition, as well as changes in gut microbiota, can profoundly impact immune function, as a vast majority of immune cells are located in the gut *lamina propria* (41). Significant advances have been made in recent years to understand the impact of MACs on the immune system. However, much of the focus has been on high-MAC diets with little insight on the impact of MAC deprivation on the immune system. A comprehensive survey of MAC enrichment vs. deprivation is essential for deciphering how specific immune pathways are regulated under varied dietary habits, and whether MACs plays a preventative or corrective role in various diseases.

### Dendritic Cells

Short-chain fatty acids elicit numerous effects on the function and hematopoiesis of dendritic cells. Propionate was shown to alter DC precursors in the bone marrow, which attenuated their ability to promote Th2 effector cells in the lungs (32). Indeed, mice fed on a low-MAC diet developed exacerbated allergic airway inflammation.

Short-chain fatty acid, particularly acetate and butyrate, could also affect tolerogenic DC function (22). Under no-MAC feeding conditions, CD103^+^ dendritic cells had a diminished ability to generate a tolerogenic regulatory T cell (Treg), compared to high-MAC feeding conditions. Mice fed on a no-MAC diet exhibited severe clinical anaphylaxis compared to mice fed on a high-MAC diet in a model of food allergy. Protection by high-MAC diet was mediated through the enhancement of retinal-dehydrogenase activity in CD103^+^ dendritic cells, an enzyme required for the conversion of vitamin A into retinoic acid to promote Treg differentiation. As such, the protective effects of high-MACs on food allergy were abrogated in the absence of vitamin A in diet. Both the SCFA receptors such as GPR43 and GPR109A were indispensable for high-MAC-mediated protection against food allergy. Interestingly, mice fed on a control MAC diet (equivalent to recommended amount of 14 g/1,000 kcal/day) exhibited similar severity to food allergy to mice fed on a no-MAC diet. This suggests that, in this instance, high levels of MACs are necessary for optimal protection against development of food allergies rather than the recommended amount (22).

However, despite these beneficial effects of SCFAs in allergy, the role of SCFAs in tolerance is not clear; as a recent study has highlighted both beneficial and detrimental effects of SCFAs in experimental autoimmune diseases and antibody-induced arthritis, respectively (42). The mechanisms behind these differential effects in autoimmunity are yet to be determined.

### T Cells

Short-chain fatty acids have a broad impact on T cell function by directly promoting the differentiation of naïve T cells into Treg (23, 43, 44), Th1, and Th17 (45) and indirectly inhibiting Th2 differentiation (22).

Acetate has been shown to promote Treg differentiation by inhibiting the histone deacetylase HDAC9 in T cells, stimulating transcription of Foxp3 (5). This was a key mechanism in the protection against allergic airway inflammation as mice fed on no-MAC diet developed exacerbated disease. Treatment with acetate was also protective independently of GPR43, suggesting that the beneficial effects of MACs were solely based on HDAC inhibition.

Both acetate and butyrate promote the induction of Th1 and Th17 T cells. These effects were also dependent on HDAC inhibition and activation of the pathway mTOR–ribosomal protein S6 kinase, but independent of GPR41 and GPR43 signaling (45). Induction of Th1, Th17, and IL-10-producing T-cells by acetate and butyrate might be key in the beneficial effects of high MACs in *C. rodentium* infections (46).

### B Cells

Microbiota-accessible carbohydrates appear to be pivotal regulators of antibody response both locally in the gut and systemically as mice fed on an MAC-deficient diet have defective homeostatic and pathogen-specific antibody responses (31). In contrast, mice fed on a high-MAC diet had significantly enhanced IgA production compared to mice fed a no-MAC diet (22, 31). High-MAC feeding increased T follicular helper response marked by increased germinal center activities in the Peyer’s patches, as well as IgA^+^ B cells in the small intestine (22). These effects on B cells are both linked to MAC-induced changes in the gut microbiota composition (22) and to the production of SCFAs as they support B cell antibody production by promoting plasma cell differentiation (31). Kim et al. also demonstrated that MAC deficiency increased *C. rodentium* disease burden due to reduced antibody production and diminished clearance of *C. rodentium*. Deficiency in dietary MACs may therefore alter B cell response and predispose to bacterial infection burden.

### Neutrophils

Dietary MACs were first linked to leukocyte migration by identification of SCFA receptor GPR43 as a neutrophil chemoattractant (47, 48). SCFAs were shown to elicit GPR43-dependent activation of PKB, p38, and ERK in neutrophils leading to their migration through polycarbonate filters toward a source of acetate, propionate, or butyrate (48). On the other hand, absence of GPR43 exacerbated recruitment of neutrophils in DSS-induced acute colitis, chronic colitis, and in a model of systemic LPS challenge (47, 49, 50), suggesting differential roles for GPR43 on neutrophil migration under inflammatory vs. non-inflammatory conditions.

It has also been shown that a low-MAC diet exacerbated gout in a mouse model and that acetate promoted resolution of neutrophilic inflammation in a GPR43-dependent manner. This resolution of inflammation was associated with increased caspase-mediated neutrophil apoptosis in MSU-challenged mice (51). Thus, deficiency in MACs might not only exacerbate inflammatory reaction but also impair inflammatory resolution.

## Conclusion

Food processing has significantly decreased the amount of MAC content in the Western diet, when compared to the contents of the ancestral hominin diet (7). As discussed above, low-MAC consumption not only has detrimental impacts on gut microbiota in particular but also on the host as a whole. It favors the development of diseases and increases mortality, as shown in preclinical and clinical studies (5, 9, 52). Low consumption of MACs over generations leads to the complete disappearance of beneficial bacterial strains in a preclinical study (26). While such study has not been carried out in humans, it is known that, similar to mice, the consumption of a diet low in MACs decreases bacterial diversity. This seems to suggest that long-term-reduced MAC consumption over generations will likely also have detrimental effects in humans. The only treatment able to correct this “scarred microbiota” was combined dietary and probiotic interventions. This might explain the low efficacy of probiotic-exclusive treatments in humans, as probiotics may not grow in a dysbiotic environment. Thus, dietary interventions alongside administration of beneficial bacterial strains could be a cost effective treatment to manage most non-communicable Western lifestyle diseases. Extensive research still needs to be done to determine (1) what types of MACs are most efficient at diversifying the microbiota and promoting production of SCFAs and (2) how much MACs should be consumed to optimize maintenance of health, or to treat different types of inflammatory diseases.

## Author Contributions

The review was cowritten by CD, GP, JT, and LM.

## Conflict of Interest Statement

The authors declare that the research was conducted in the absence of any commercial or financial relationships that could be construed as a potential conflict of interest.

## Abbreviations

DSS, dextran sodium sulfate; MACs, microbiota-accessible carbohydrates; SCFAs, short-chain fatty acids.
